# Case Report: A case of *Nocardia otitidiscaviarum* pneumonia diagnosed by application of metagenome next-generation sequencing and a narrow literature review

**DOI:** 10.3389/fmed.2025.1646940

**Published:** 2025-11-18

**Authors:** Miao Kong, Jiaying Sun

**Affiliations:** Department of Pulmonary and Critical Care Medicine, The Fourth Affiliated Hospital of China Medical University, Shenyang, China

**Keywords:** *Nocardia otitidiscaviarum*, pulmonary infection, clinical features, metagenome next-generation sequencing, treatment

## Abstract

*Nocardia* is an opportunistic pathogen with relatively low incidence but high mortality. Recently, reports of *Nocardia* infections have increased; however, infections caused by *Nocardia otitidiscaviarum (N. otitidiscaviarum)* remain relatively rare. Due to its non-specific clinical manifestations and imaging features, *N. ototidiscaviarum* infections are frequently misdiagnosed or underdiagnosed, and no standardized guidelines currently exist for their diagnosis and treatment. In this study, we report a case of pulmonary infection caused by *N. otitidiscaviarum*, which was diagnosed using a combination of traditional microbial morphology and second-generation sequencing, and subsequently showed improvement following treatment with trimethoprim-sulfamethoxazole (TMP-SMZ) and linezolid. Additionally, we conducted a comprehensive literature review using PubMed to provide insights for improving the diagnosis and treatment of *N. otitidiscaviarum* infections.

## 1 Introduction

*Nocardia* is a genus of gram-positive aerobic actinobacteria widely distributed in soil, decaying organic matter, and stagnant water (1). It is an opportunistic pathogen, with most species exhibiting weak positivity in acid-fast staining. *Nocardia* typically invades the body through the respiratory tract, skin, mucosal surfaces, or bloodstream, leading to localized or systemic disseminated infections, with primary lung infection being the most common manifestation (2). *Nocardia* infections have a low incidence but a high mortality rate. Among these, *Nocardia otitidiscaviarum (N. otitidiscaviarum)* is relatively uncommon, accounting for only 0.3%−2.9% of cases (3). Historically, only a limited number of case reports have been documented in both domestic and international literature; however, recently, the number of reported cases has increased. Due to its slow growth and challenges in culture and isolation, the culture process is frequently inhibited by commensal bacteria, and traditional microbial morphology detection methods are prone to diagnostic errors. Additionally, the lack of standardized guidelines for diagnosis and treatment further complicates clinical management. Here, we present a case of *N. otitidiscaviarum* pneumonia diagnosed using a combination of traditional microbial morphology and second-generation sequencing. Furthermore, we summarized and analyzed the clinical characteristics, diagnostic approaches, and treatment strategies based on a PubMed literature review to enhance the understanding and management of *N. otitidiscaviarum* infections.

## 2 Case presentation

A 53-year-old male technician was admitted to the respiratory internal medicine department on March 18, 2024, due to fever and shortness of breath on exertion for 10 days. The patient reported an onset of high fever (maximum 40 °C) without an obvious cause before admission. Symptoms were accompanied by chills, dyspnea on exertion, production of a small amount of yellow sputum, and mild right-sided chest pain during deep inspiration. He initially self-medicated with oral cephalosporin. A local central hospital diagnosed him with a pulmonary mass suggestive of malignancy and pneumonia, and treated him with cephalosporins and antiviral drugs for 1 week, without clinical improvement. Subsequently, he was admitted to our hospital for further consultation and treatment. His medical history included hypertension and a history of an open toe fracture 1 year before presentation. Findings on admission revealed a temperature of 39 °C, pulse of 104 beats/min, respiration of 31 beats/min, blood pressure of 130/82 mmHg, shortness of breath with coarse breath sounds bilaterally, and wet rales noted in the right lung. No other significant abnormalities were observed.

**Figure 1:**
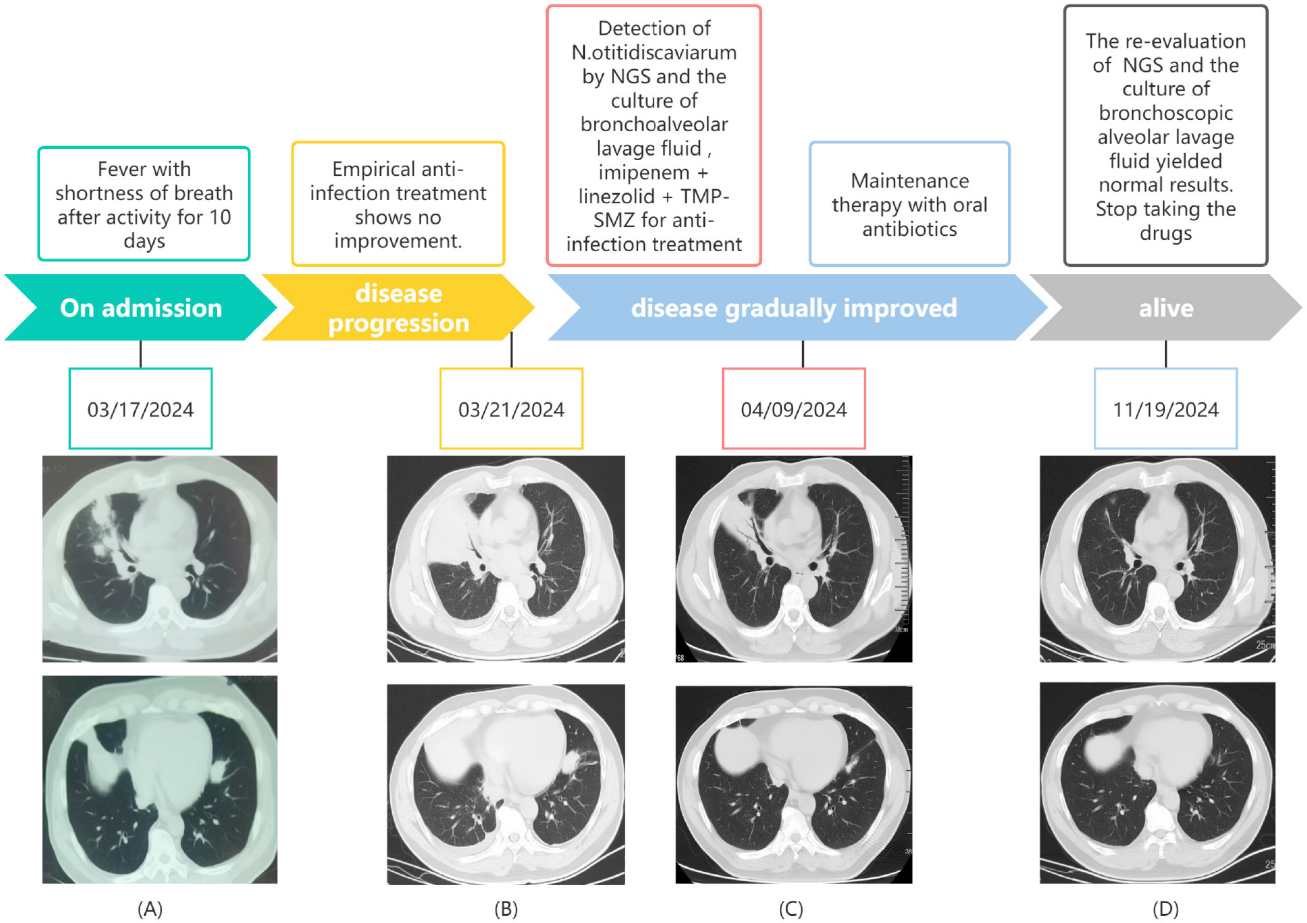
The timeline of the patient's disease progression and treatments. **(A)** At the onset of the illness; **(B)** 4 days after admission; **(C)** 22 days after admission; **(D)** 8 months after admission.

Chest computed tomography (CT) (March 17) revealed a mass-like soft tissue density shadow in the middle lobe of the right lung (87 × 71 mm), with irregular margins and increased density in adjacent lung tissues. Multiple spherical hyperdense shadows were observed in the left lung, lower lobe of the right lung, and upper lobe of the right lung, along with an enlarged lymph node (see Figure 1A). An electrocardiogram performed on March 18 revealed sinus tachycardia. Arterial blood gas analysis revealed pH, PaO_2_, PaCO_2_, and PaO_2_/FiO_2_ of 7.47, 57, 24.8, and 271 mmHg, respectively. Blood count revealed elevated leukocyte and neutrophil levels, along with an increased neutrophil ratio. Biochemical tests showed elevated urea, fasting blood glucose, lactate dehydrogenase, and glycated hemoglobin. Infection markers such as erythrocyte sedimentation rate (ESR), high-sensitivity C-reactive protein (CRP), calcitonin, interleukin-6 (IL-6), and ferritin were elevated. Tumor markers showed no significant abnormalities. Immunological indicators included decreased absolute counts of CD4^+^ lymphocytes and natural killer (NK) cells (see Table 1). Liver function, cardiac enzymes, troponin, NT-proBNP, and tumor markers were within normal limits. Pathogenetic tests, including respiratory virus antibody assays, β-D-glucan/endotoxin, Legionella and tuberculosis antibodies, Mycoplasma/Chlamydia IgM, and sputum smears (bacterial, fungal, and acid-fast), were all negative.

**Table 1:** Patient's laboratory findings.

**Laboratory findings**	**DAY 1**	**DAY 17**	**Normal range**
WBC (× 10^9^/L)	15.71	6.36	4–10
Neutrophile granulocyte (× 10^9^/L)	13.86	3.98	1.9–8
Neutrophile granulocyte percentage (%)	88.2	62.6	84.2
Blood urea nitrogen (mmol/L)	7.5	5.39	3.2–7.1
Fasting plasma glucose (mmol/L)	8.52	7.1	4.1–5.9
Lactate dehydrogenase (U/L)	334	234	120–246
Erythrocyte sedimentation rate (mm/h)	96	20	0–15
Procalcitonin (ng/ml)	0.668	0.071	0–0.046
C-reactive protein (mg/L)	375	9.55	0–5
Interleukin 6 (pg/ml)	251.8	12.7	0–7
Fibrinogen (g/L)	8.28	3.91	2–4
D-dimer (ng/ml)	1,004	200	0–252
HIVDNA	Negative	–	Negative
Cluster of differentiation 4+	253	–	410–1,590
Cluster of differentiation 8+	384	–	190–1,140
Natural killer cell	68	–	90–590
Oxygenation index (mmHg)	271	338	400–500

After admission, empirical intravenous anti-infective treatment was initiated with cefoperazone sodium-sulbactam sodium combined with omadacycline mesylate. Transnasal high-flow oxygen therapy was administered to improve oxygenation, and supportive treatment to enhance immunity was provided. The patient was placed on a diabetic diet with adequate energy intake for glycemic control. Vital signs and oxygen saturation were closely monitored. Enhanced chest CT revealed multiple patchy, clustered, cord-like, and solid opacities in the bilateral lower lobes (see Figure 1B). Focal air bronchograms and small air vacuoles were noted in the right middle lobe, which showed heterogeneous enhancement on contrast scanning. Other lesions appeared more uniformly intensified. Based on these findings, pneumonia with lung abscesses was considered the most likely diagnosis.

After 3 days of persistent fever, bronchoscopic alveolar lavage (BALF) was performed; the specimen was sent for bacterial and fungal smear and culture, and metagenomic next-generation sequencing (mNGS) was immediately conducted.

Briefly, mNGS detection was performed as follows (details are provided in the Supplementary material): DNA was extracted using a nucleic acid extraction and purification kit (Genskey, Tianjin, China). The extracted nucleic acids were subjected to fragmentation, end repair, adapter ligation, PCR amplification, and magnetic bead purification to construct the DNA library. Library quality control was performed using an Agilent 2100 Bioanalyzer (Agilent Technologies, Santa Clara, USA) to assess library and insert fragment sizes. The constructed library was pooled and sequenced on the MGISEQ-2000 platform (MGI Tech Co., Ltd, Shenzhen, China). Subsequently, low-quality, low-complexity, and short sequences were filtered out using fastp software. Human DNA sequences were removed by aligning with bowtie2 against the human reference genome T2T-CHM13 (Telomere-to-Telomere Consortium Human Genome Assembly in Chromosome 13). The resulting high-quality sequencing data were aligned against a custom reference database using the BWA-MEM software tool. This database was primarily constructed from the NCBI RefSeq and GenBank genomic databases.

**Figure 2:**
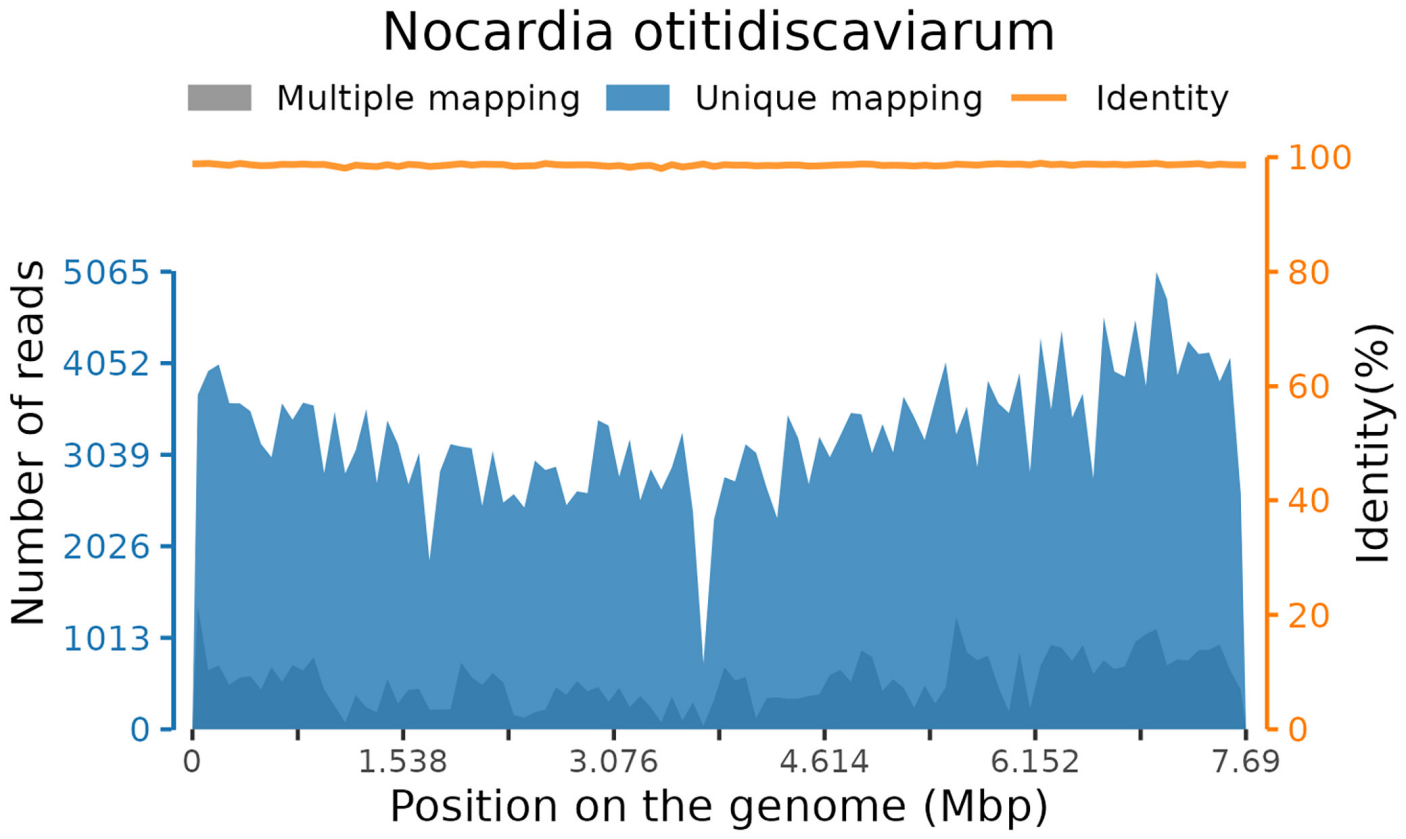
Coverage of *Nocardia otitidiscaviarum* detected by mNGS in BALF.

**Figure 3:**
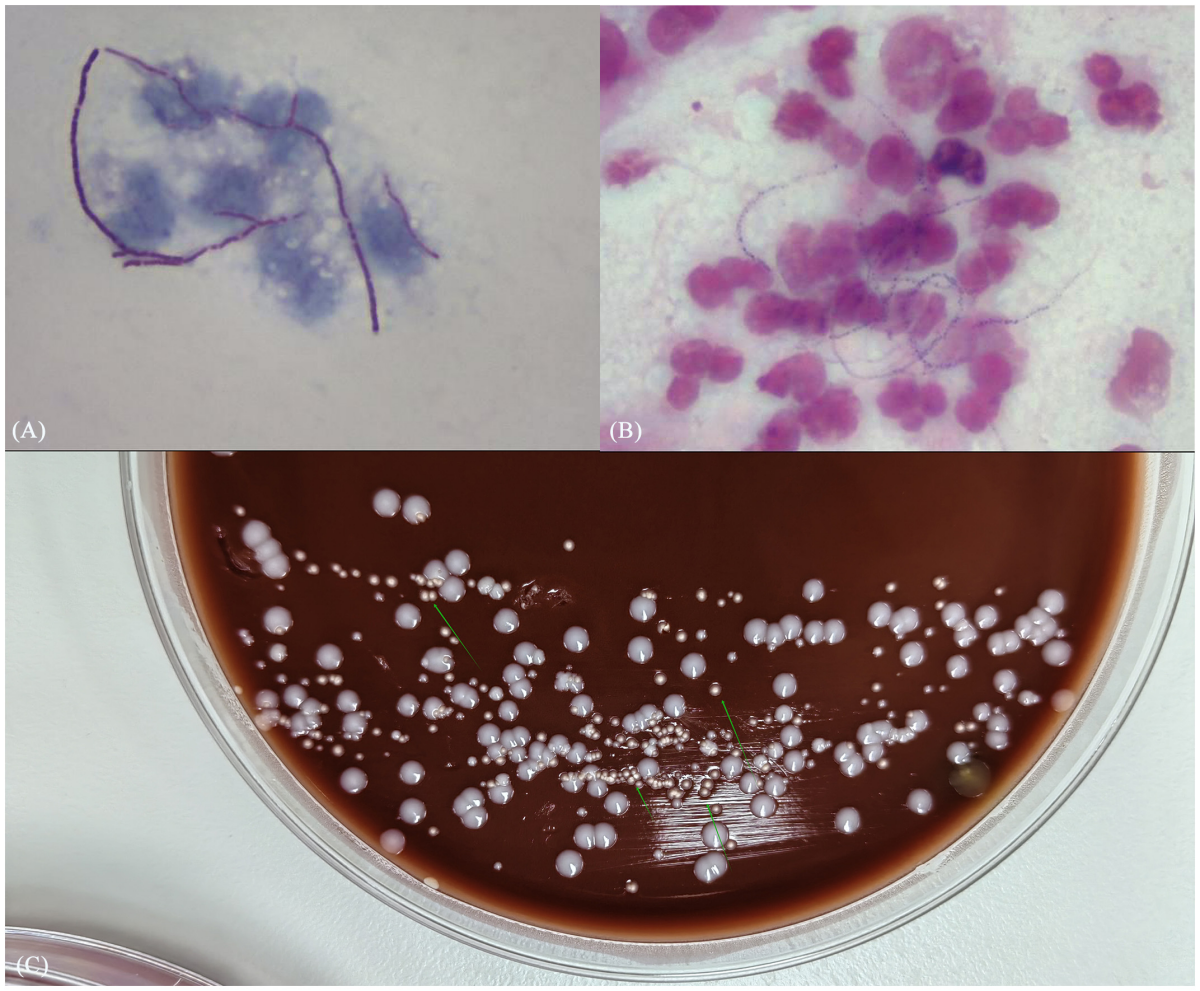
**(A)** Gram stain smear of alveolar lavage fluid showed weakly positive, bead-like hyphae; **(B)** weak acid-fast staining revealed dendritic red hyphae; **(C)** culture results identified pale white dry colonies.

Subsequently, mNGS of the BALF identified a rare pathogen, *N. otitidiscaviarum*, with 327, 542 sequencing reads and 74.41% coverage (see Figure 2). Additionally, this pathogen was successfully cultured from the BALF (see Figure 3). The thin-layer cytology of the BALF revealed no evidence of malignant tumor cells.

On post-admission day 5, the anti-infective regimen was adjusted to include oral trimethoprim-sulfamethoxazole (TMP-SMZ) and linezolid combined with imipenem-cilastatin sodium. To clarify the presence of disseminated infection, head and whole abdominal CT was performed; no obvious abnormality was observed. On day 7, body temperature normalized, dyspnea improved, and the oxygenation index showed significant improvement. On day 22, chest CT revealed partial absorption of lung lesions, with cavity formation in some areas (see Figure 1C). The anti-infective regimen was adjusted to oral TMP-SMZ and linezolid for maintenance therapy. On day 25, the patient was discharged without apparent discomfort. Post- discharge follow-up, the patient alternated weekly courses of oral TMP-SMZ and linezolid tablets. At the 8-month follow-up, the patient was re-hospitalized for review of NK cell count, which remained at a low level; however, repeat BALF showed no abnormalities, and chest CT demonstrated significant lesion absorption (see Figure 1D). Based on this finding, medication was discontinued. There was no recurrence on follow-up lung CT.

## 3 Literature review

**Figure 4:**
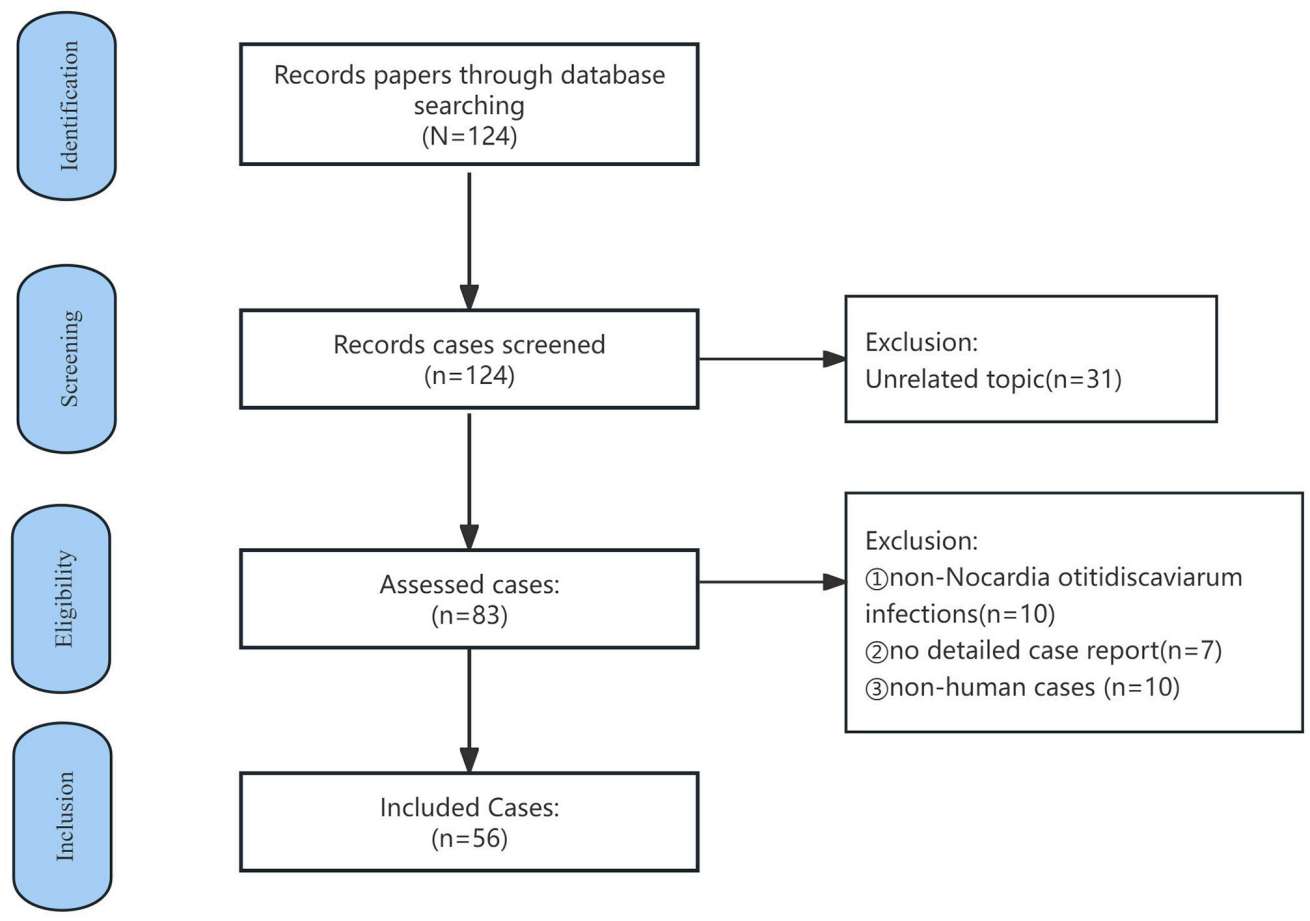
Case selection flowchart for *N. otitidiscaviarum* infection in the literature review.

A literature search was conducted in Pubmed using the keyword “*Nocardia otitidiscaviarum*” covering the period 1980–2024. Case reports lacking detailed patient information, non-*N. otitidiscaviarum* infections, and non-human studies, as well as those unrelated to *N. otitidiscaviarum* infections in humans, were excluded (see Figure 4). Demographic information, including age, sex, infection site, medical history, drug sensitivity test results and clinical outcomes, was extracted and compiled. Since this is a narrative review (rather than a systematic review), we aimed to synthesize diverse published experiences with *N. otitidiscaviarum* instead of conducting a formal meta-analysis. Statistical analyses were performed using SPSS version 26. Categorical variables were analyzed using chi-square tests or Fisher's exact tests. Continuous variables underwent *t*-test. Age was reported using mean ± standard deviation. Statistical significance was defined as *p* ≤ 0.05. Ultimately, 56 cases were included in this study (Table 2 and Supplementary Table S1 provided a summary of the features of all the included studies), all of which were case reports.

**Table 2:** Clinical characteristics of 56 cases of *N. otitidiscaviarum* infection.

**Characteristics**	**All patient (*N* = 56)**	**Cured (*N* = 41)**	**Treatment failure (*N* = 15)**	**Univariate analysis**
				***P*** **Value**
Male	38	28	10	1.000
Age, years, median (Interquartile range)		55.46 ± 18.24	55.13 ± 20.70	0.954
**Geographic distribution**				0.704
Asia	35	26	9	
Africa	1	1	0	
Europe	12	7	5	
North America	7	6	1	
South America	1	1	0	
**Confirmed time**				0.183
After the year of 2010	37	25	12	
Before the year of 2010	19	16	3	
Certain risk factor	48	34	14	0.428
Underlying disease	43	29	14	
Trauma	7	7	0	
Intravenous history	1	1	0	
PICC	1	1	0	
Immunosuppressive therapy	20	15	5	0.822
**Infection site**				0.045
Lung	22	14	8	
Skin/Lymph node	12	12	0	
Brain	3	3	0	
Disseminability	19	12	7	
**Diagnostics**				0.463
Microbiology	31	21	10	
Molecular biology	3	2	1	
Both	22	18	4	
**Drug sensitivity test**				0.070
Sensitive to TMP-SMZ	30	23	7	
Resistant to TMP-SMZ	11	5	6	
None	15	13	2	
**Treatment time**				0.000
≥6 months	22	22	0	
3–6 months	13	12	1	
<3 months	19	5	14	
Unknown	2	2	0	

## 4 Discussion

### 4.1 Pathogen and epidemiology

*Nocardia* is an opportunistic, non-commensal bacterium that typically invades the body via the skin and mucous membranes, bloodstream, or respiratory tract, leading to localized or systemic infections that are either pyogenic or granulomatous (4). The lungs are the most frequently affected organ, accounting for approximately 60%−70% of all human *Nocardia* infections (5), primarily resulting from inhalation of spores or bacterial fragments (6). In addition to pulmonary involvement, *Nocardia* can infect various organs, including the skin and brain, and can result in systemic dissemination and sepsis, which may be life-threatening (7). The prognosis for Nocardiosis is poor, and the literature indicates that 54 of the 119 validly named *Nocardia* spp. are associated with human infections, with the most frequent isolates, including *Nocardia brasiliensis, Nocardia farcinica, Nocardia abscessus* complex*, Nocardia nova* complex*, Nocardia cyriacigeorgica* and *Nocardia transvalensis* complex (8). Wang reported that *N. otitidiscaviarum* accounted for 5.9% of the 441 *Nocardia* infection cases (8). *N. otitidiscaviarum* was originally isolated by Snijders in 1924 from the diseased middle ear of guinea pigs or Sumatran house mice (9). The first documented human infection was not reported until the 1960s, involving two cases of disseminated infection that ultimately resulted in death (10). Although *Nocardia* infections are gradually being recognized, *N. otitidiscaviarum* has a lower incidence rate than other species of the *Nocardia* genus, primarily due to factors, such as reduced virulence and slower growth in soil environments. Reports on this species are relatively rare; however, its mortality rate remains relatively high (7), with a mortality rate of approximately 27%, as presented in Table 2. Conversely, this significant mortality risk has not been sufficiently emphasized, making accurate diagnosis and treatment even more challenging (11). Clinically, *N. otitidiscaviarum* infections can be categorized based on the site of infection, such as pulmonary, central nervous system, skin, subcutaneous, or disseminated infection (12). In our review of the literature (Table 2), although no significant differences in disease outcomes were observed between sexes, the total number of infected individuals showed a clear male predominance (38 males vs. 18 females), consistent with the findings summarized by Rawat et al. (13), this may be related to hormonal effects on *Nocardia* virulence or growth. Notably, 37 of the 56 cases (66%) included in our review were published in the past 15 years, suggesting a growing clinical awareness and concern regarding *N. otitidiscaviarum* as a pathogen of emerging clinical significance.

### 4.2 Risk factors

Most individuals infected with *N. otitidiscaviarum* have underlying structural lung diseases or immunodeficiency conditions, including long-term use of immunosuppressants or glucocorticoids, stem cell and solid organ transplantation, chronic kidney disease, malignant tumors, chronic lung disease, smoking and alcoholism (14). However, up to 15% of *Nocardia*-associated infections occur in immunocompetent individuals (15). Among the 56 cases summarized in Table 2, 48 cases (85.7%) had clearly defined risk factors or immunosuppressive conditions. Respiratory comorbidities ranged from common conditions (e.g., chronic obstructive pulmonary disease, asthma and bronchiectasis) to less common ones (e.g., tuberculosis, cystic fibrosis, and chronic thromboembolic pulmonary hypertension). Non-respiratory diseases included underlying malignancies, organ transplantation, autoimmune diseases, diabetes, long-term dialysis, and HIV infection. Notably, 8 (14.2%) cases in Table 2 involved immunocompetent individuals with no underlying disease. Nocardiosis primarily affects immunocompromised patients with impaired T cell-mediated immunity (16). However, animal models have identified immune factors contributing to *Nocardia* susceptibility and their underlying mechanisms, while the immunological characteristics of nocardiosis in humans remain largely unelucidated (17), necessitating further research. NK cells play a crucial role in innate immunity, recognizing and attacking viruses and bacteria. Severe infections are associated with significantly lower NK cell counts (18). Persistent NK cell dysfunction contributes to infection-induced immunosuppression, increasing susceptibility to secondary infections or reactivation of latent viruses, which can lead to poor prognosis (19). Various primary immunodeficiency diseases can involve NK cells, resulting in NK cell defects, which lead to susceptibility to pathogenic bacteria. In this case, the patient had an open toe fracture 1 year prior, which developed suppurative complications and had remained unhealed before working outdoors for approximately 6 months. The infection pathway may have involved contact with contaminated soil or water sources. Susceptibility factors were considered after the patient was admitted to the hospital: laboratory tests revealed high blood glucose levels, immunological results showed a reduced T-lymphocyte subpopulation, and repeated laboratory tests confirmed a reduced NK cell proportion. Despite no history of HIV, repeated retests were not abnormal; however, the patient exhibited deficiencies in both NK cell-mediated innate and CD4^+^ T lymphocyte-mediated acquired immunity. These findings indicate that the patient had multiple predisposing factors, which increased susceptibility to *N. otitidiscaviarum* infection. However, the possibility of an underlying immunodeficiency disease warrants further follow-up and long-term monitoring.

Previous studies have indicated that *N. otitidiscaviarum* is primarily transmitted by inhaling mycelial fragments into the respiratory tract or direct invasion through the skin and mucous membranes. No documented cases of human or animal-to-human transmission exist (4). Among the 56 cases analyzed in our review (Table 2), 7 (12.5%) involved skin or mucosal breaks due to trauma, intravenous drug use, or Peripherally Inserted Central Catheter (PICC) placement, increasing susceptibility to infection. *N. otitidiscaviarum* is commonly found in decaying organic matter, garden soil, house dust, beach sand, and swimming pools (20). Individuals who work in various outdoor occupations or participate in recreational activities are at a high risk of exposure. Given the patient's history of outdoor work, environmental contact may be a risk factor for infection. Most cases summarized in Table 2 were from Asia, Europe, and North America (*n* = 54, 96.4%), which may be due to shared Mediterranean climate conditions, as reported by Shen et al. (21). Asia has the highest prevalence due to more agricultural and soil-related work. Tropical regions, including Southeast Asia, are exposed to decaying soil and organic matter, further increasing the risk of infection (22).

### 4.3 Clinical characteristics

*N. otitidiscaviarum* infections pose a diagnostic challenge due to their lack of specific clinical and imaging features, which can easily lead to misdiagnoses. Pulmonary infection symptoms include fever, cough, sputum, and shortness of breath. Laboratory findings included elevated leukocyte counts, CRP, and other indicators of infection, as well as decreased lymphocyte counts and hypoalbuminemia (23). From an imaging perspective, pulmonary infections caused by *N. otitidiscaviarum* frequently manifest as consolidation, nodules, and cavitary lesions. Less commonly, lung abscesses and pyothorax may be observed. Several studies have suggested that pleural involvement occurs due to direct extension from the chest wall and the lung parenchyma, leading to these lesions (24). Additionally, hilar lymphadenopathy or combinations of these radiological patterns can make it challenging to distinguish *N. otitidiscaviarum* infections from other diseases, including bacterial, fungal, and tuberculosis-related infections, as well as non-infectious diseases such as lung tumors (25).

### 4.4 Diagnosis

Early identification of *N. otitidiscaviarum* is crucial for accurate diagnosis; however, current methods face significant challenges. No serological tests exist for rapid detection, making microbiological tests, including smear and culture of sputum specimens, abscesses, wound drainage, or bronchial lavage fluid, the primary diagnostic tools. Microscopic visualization of gram-positive branching filaments remains a key diagnostic indicator (26). However, due to its slow growth, the traditional culture methods may take 1 week or longer (27) and biochemically differ from other *Nocardia* species in their ability to hydrolyze hypoxanthine and xanthine, further complicating routine clinical diagnosis (28). Additionally, the pathogen's routine biochemical reactions are non-specific, and its staining and culture characteristics are similar to those of to *Mycobacterium tuberculosis*, making traditional microbiological detection methods less effective. Futhermore, most hosts of nocardiosis may have received antibiotic treatment before sample collection, which reduces the sensitivity of microbial culture, thereby increasing the risk of clinical underdiagnosis, misdiagnosis, and delayed treatment. *N. otitidiscaviarum* is associated with low morbidity but high mortality in infected individuals and can cause rapidly progressive infections; Deepa reported a case of a 14-year-old patient with *N. otitidiscaviarum* pneumonia who died before the diagnosis was made (24). Moreover, it has been documented that *N. otitidiscaviarum* is susceptible to mixed infections with other pathogens (29, 30). Therefore, it is important to be cautious to this infection and implement targeted strategies for early detection to prevent diagnostic errors and ensure timely treatment. Among the 56 cases summarized in Table 2, traditional microbiological testing was used in 31 cases (55.3%), while molecular testing methods (e.g., gene sequencing, mass spectrometry) were used in 25 cases (44.6%). Currently, molecular techniques, such as mass spectrometry, gene sequencing, and NGS, offer improved accuracy. However, polymerase chain reaction (PCR)-based genetic testing requires the design of specific primers or probes, which poses challenges for detecting unknown pathogens or those with genetic mutations. Simultaneously detecting multiple pathogens using this approach is difficult, making it potentially less suitable for cases of mixed infections. Although NGS is widely used in clinical practice, particularly for infectious diseases-due to its high efficiency and accuracy (31)-it also increases healthcare costs. The slow-growing nature of *Nocardia* also prevents timely culture of target colonies, resulting in a prolonged reporting timeframe. Traditional microbial morphological testing remains indispensable, as it enables immediate sample collection from the infection site and rapid provision of pathogenic evidence, such as smear staining, in the shortest possible time, suggesting that traditional microbial morphology testing remains indispensable. Therefore, in clinical practice, the combination of traditional microbial morphology testing and NGS is required for a rapid, comprehensive, and accurate early diagnosis.

### 4.5 Treatment

Accurate identification of *Nocardia* species is the first step in treatment, as antibiotic resistance varies significantly among strains (8). Therefore, species identification and antimicrobial susceptibility testing are required before initiating antimicrobial therapy. The choice of anti-infectious drugs against *Nocardia* is relatively limited. *N. otitidiscaviarum* is resistant to β-lactam antibiotics such as ampicillin and amoxicillin-clavulanic acid, and TMP-SMZ remains the first-line treatment for *N. otitidiscaviarum* (32). However, some differences exist in the drug sensitivity of various bacterial strains, and a gradual increase in drug resistance has been reported (3). Antimicrobial susceptibility testing was performed in 41 of the 56 included cases (73.2%; Table 2). Of these 41 cases, 30 (73.1%) were susceptible to TMP-SMZ and 11 (26.9%) were resistant to sulfonamides. Other agents, including aminoglycosides (e.g., amikacin), linezolid, quinolones, and minocycline, have been used as second-line treatments (14). A study investigating *Nocardia* infections in China from 2009 to 2021 revealed that all isolated *Nocardia* strains were susceptible to linezolid, followed by amikacin (99.3% susceptibility) and TMP-SMZ (99.1% susceptibility);among the strains resistant to TMP-SMZ, all were *N. otitidiscaviarum* (8). However, an increased risk of abnormal hematologic markers after 4 weeks of linezolid administration has been reported in the literature (33), therefore, further attention is required. Additionally, β-lactams such as imipenem are sometimes used as an alternative treatment to TMP-SMZ. However, imipenem has shown different in vitro activities against various *Nocardia* spp. in numerous studies. Therefore, drug sensitivity testing is necessary.

Current treatment guidelines for *N. otitidiscaviarum* infections recommend monotherapy for cutaneous infections, stable pulmonary disease and patients with mild illness. However, for severe infections (e.g., severe pulmonary infection, central nervous system involvement, and disseminated disease), especially in immunosuppressed patients, at least two agents are recommended as initial therapy (34). Common regimens include TMP-SMZ + amikacin + minocycline or imipenem, administered for at least 3–4 weeks, followed by sequential monotherapy (35). The literature suggests that linezolid, either alone or in combination with sulfonamides, can be effective in treating moderate to severe nocardiosis (36). Amikacin is rarely used as monotherapy due to its limited penetration into certain infection sites (such as the central nervous system) and some potential toxicities. In this case, because of laboratory limitations, antimicrobial susceptibility testing was not performed. After diagnosis to avoid delaying treatment, empirical initial therapy with cotrimoxazole, linezolid and imipenem-cilastatin sodium was based on literature evidence. Following clinical improvement, the patient was discharged and switched to oral linezolid tablets and TMP-SMZ. In addition to oral or intravenous antimicrobial therapy, adjunctive treatments-including abscess incision and drainage, intrathecal antibiotic injection, surgical resection of lesions, and improvement of the immunization regimen-should be considered in combination when necessary (37).

Treatment for *Nocardia* should be thorough due to the high recurrence rate (35). While the optimal duration for treating *N. otitidiscaviarum* infections remains uncertain, most literature recommended that at least 6 weeks of therapy for localized infections in immunocompetent patients. Some experts have reported that recurrence is rare in individuals who have received treatment for more than 3 months (38). In Table 2, 82.9% of the patients who showed clinical improvement had received treatment for more than 3 months. At least 6 months of treatment is recommended for disseminated infections, and at least 1 year of treatment is recommended for immunocompromised patients and those with central nervous system infections (39). Ensuring complete resolution of the lesion via imaging before discontinuing antimicrobial therapy. Treatment for nocardiosis should be initated as early as possible. In patients with a high clinical suspicion of *Nocardia* infection, particularly in immunocompromised individuals such as organ transplant recipients, empirical therapy should be initiated promptly before susceptibility testing results are available (34), and the need for prolonged therapy or prophylaxis should be determined based on the patient's underlying condition and immune status.

### 4.6 Prognosis

Clinical outcomes of patients with *N. otitidiscaviarum* infections are influenced by multiple factors. Among the cases included in our literature review (see Supplementary Table S1), 15 deaths were reported (accounting for 26.7% of the 56 included cases). Further analysis identified several factors associated with poor prognosis, including delayed diagnosis (*n* = 1), co-infection with other pathogens (*n* = 1), severe underlying diseases (*n* = 5), and failure to perform timely antimicrobial susceptibility testing or sulfonamide resistance leading to inappropriate treatment (*n* = 8). There were two cases of recurrence, one was due to disseminated infection plus delayed testing, and the other was due to an insufficient antimicrobial regimen. Therefore, high clinical severity, secondary infections, high-risk clinical severity, secondary infections, presence of risk factors and comorbidities, and delay in diagnosis and treatment were positively associated with poor prognosis (3).

## 5 Conclusion

In conclusion, *N. otitidiscaviarum* infections are relatively underreported, associated with a high mortality rate, and require early detection for prompt intervention. Therefore, the accurate identification of the species is critical for timely diagnosis and treatment. When standardized anti-infection measures prove ineffective, the possible presence of *Nocardia* should be noted, and specimens should be sent for testing promptly. Laboratory diagnostics play a vital role, with BALF analyzed through NGS and traditional microbial morphology testing aiding in rapid pathogen identification. Early standardized anti-infective treatment is key to successful recovery from *N. otitidiscaviarum* infection.
