# Supplementary material for: Case Report: A case of Nocardia otitidiscaviarum pneumonia diagnosed by application of metagenome next-generation sequencing and a narrow literature review
Source: Front Med (Lausanne). 2025 Nov 18;12:1646940. doi: 10.3389/fmed.2025.1646940 (PMC12669003; doi:10.3389/fmed.2025.1646940)
Supplement: Supplementary file 1 [file Table_1.docx]

**Supplementary Material**

**Table S1.**Studies reviewed, including patient characteristics,diagnostic methods,

antimicrobial therapy and drug susceptibility profile.

| Authors/year of  publication | Geographic distribution | Age and gender | Risk Factors | Infection site | Diagnostics | Treatment | Drug Susceptibility | Outcome | Treatment Time |
| --- | --- | --- | --- | --- | --- | --- | --- | --- | --- |
| Simmons et al,1992(Simmons et al., 1992) | America | 60M | heart transplant | Skin, soft tissue, lung | culture | Imipenem/cilastatin, TMP-SMZ and doxycycline 28 days. Doxycycline for another 2 months | Susceptible to Ciprofloxacin,Amikacin, tetracycline,imipenem  Resistant to TMP-SMZ,methoxazole,cefuroxime, cefoxitin ,amoxicillin/clavulanate | Recovered | 2.5 months |
| Yang et al,1993(Yang et al., 1993) | China | 36M | Chronic obstructive pulmonary disease, Cushing's disease, glucocorticoids | Subcutaneous, soft tissue | Pus culture | TMP-SMZ | - | Recovered | 5 months |
| Castelli et al., 1994(Castelli et al., 1994) | Italy | 31M | AIDS, intravenous drug use | Initially subcutaneous and soft tissue, 2 months later it spread to the brain | Culture of purulent secretion | TMP-SMZ, netilmicin for 20 days | - | Recurrence and death occurred two months later. | 2 months |
| Clark et al,1995(Clark et al., 1995) | America | 86M | trauma | Subcutaneous soft tissue | Culture of purulent secretion | TMP-SMZ | Susceptible to aminoglycosides, TMP-SMZ,ceftriaxone and imipenem | Recovered | 10 weeks |
| Suzuki et al,1995(Suzuki et al., 1995) | Japan | 78 F | Bronchial asthma, glucocorticoid application | Skin, soft tissue, lymph nodes | Culture of purulent secretion | Minocycline vertigo, doxycycline skin did not improve,and finally oral of ofloxacin | High Susceptible to minocycline,ofloxacin,gentamicin; moderate Susceptible to TMP-SMZ, amikacin, clindamycin; Resistant to penicillin and cephalosporin | Recovered | 3 months |
| Mereghetti et al,1997(Mereghetti et al., 1997) | France | 31M | Previous health, car accident trauma | skin | Sputum culture | TMP-SMZ and imipenem for 3 weeks, TMP-SMZ alone for 5 weeks | Resistant to penicillin,cephalosporin,quinolone,erythromycin,clindamycin,tetracycline,chloramphenicol,phosphomycin,vancomycin,tobramycin and gentamicinSusceptible to TMP-SMZ,imipenem, amikacin and kanamycin | Recovered | 2 months |
| Sandre et al,1997(Sandre and Summerbell, 1997) | Africa | 59M | AIDS | Disseminated (thoracic wall, lung, peritoneal cavity) | Suction culture of mass | Surgical debridement.  TMP-SMZ and amikacin for 6 weeks, followed by TMP-SMZ oral | Susceptible to TMP-SMZ and amikacin;  Resistant to Cefotaxime,tobramycin,gentamicin,streptomycin,minocycline and erythromycin | Recovered | Preventive lifelong medication |
| Taniguchi et al,1998(Taniguchi et al., 1998) | Japan | 76M | Tuberculosis, chronic respiratory disease | lung | Culture of lavage fluid from alveoli | Vein minocycline is effective,oral minocycline has poor response  TMP-SMZ is taken orally | Susceptible to minocycline,TMP-SMZ,bramycin,clindamycin,kanamycin and netilmicin;  Resistant to Imipenem, cloroxitin, apicillin, cefazolin, cefmetazole,cefotetan,ofloxacin, phosphomycin,roxithromycin and 5-fluorouracil | Recovered | 6 months |
| Hartmann A et al,2000(Hartmann et al., 2000) | Norway | 50F | Kidney transplantation, long-term use of glucocorticoids + immunosuppressants | brain | Sputum culture + gene sequencing | Meropenem and rifampin for 6 weeks. Ciprofloxacin and rifampin for 10 weeks. | Susceptible to Meropenem, rifampin, ciprofloxacin | Recovered | 3 months |
| Durán et al,2001(Durán et al., 2001) | Spain | 21M | Injecting drugs intravenously | brain | Tumor culture | Cefotiam and metronidazole, imipenem and TMP-SMZ 45 days improved,oral TMP-SMZ 6 months | Susceptible to aminoglycosides,TMP-SMZ,ciprofloxacin, imipenem, netilmicin;  Resistant to penicillin,second-and third-generation cephalosporins,erythromycin and vancomycin | Recovered | 7.5 months |
| Wada et al,2002(Wada et al., 2002) | Japan | 69F | Health, trauma | Skin, soft tissue | Sputum culture | TMP-SMZ | - | Recovered | 6 months |
| Alberts et al,2002(Alberts and Boyd, 2002) | America | 77 M | Rheumatoid arthritis with long-term oral glucocorticoids. Trauma | Skin, soft tissue | Biopsy and culture of calf nodules | Minocycline for 2 weeks and clarithromycin for 6 months | Moderately Susceptible to minocycline and Susceptible to clarithromycin | Recovered | 6 months |
| Dikensoy O et al,2004(Dikensoy et al., 2004) | Turkey (Asia) | 65M | Immunity is normal | lung | sputum culture | Amikacin and TMP-SMZ cased duodenal ulcer perforationImipenem, amikacin intravenous infusion; followed by TMP-SMZ oral | Only TMP-SMZ,amikacin and tobramycin were sensitive | Recovered | 6 months |
| Hemmersbach-Miller et al,2004(Hemmersbach-miller et al., 2004) | Spain | 44 M | Diabetes, kidney transplant, tuberculosis, glucocorticoids + immunosuppressants | Brain, subcutaneous, soft tissue | Drainage fluid culture | TMP-SMZ, followed by respiratory infection caused by Pseudomonas aeruginosa, pseudomembranous colitis caused by Clostridium difficile, deep vein thrombosis and progressive deterioration. | Susceptible to TMP-SMZ,gentamicin, ofloxacin, ciprofloxacin and doxycyclineResistant to cefotaxime,ceftriaxone,imipenem tobramycin,amikacin was not tested | Died | 5 months |
| Yoshida et al,2004(Yoshida et al., 2004) | Japan | 69M | Vasculitis, glucocorticoids | pyothorax | Chest water culture | Gentamicin, levofloxacin, TMP-SMZ | Susceptible to levofloxacin,gentamicin;  Moderate Susceptible to TMP-SMZ,erythromycin;  Resistant to ampicillin, piperacillin sodium, cefotetan, vancomycin | Recovered | 6  months |
| Fabre et al,2005(Fabre et al., 2005) | France | 70 M | Immunosuppressants, glucocorticoids, trauma | Subcutaneous, soft tissue | Abscess culture | Ofloxacin and clindamycin | - | Recovered | 4 months |
| Sharma et al,2007(Sharma et al., 2007) | African American | 36 F | Sickle cell anemia and end-stage renal disease | lung | blood culture | TMP-SMZ 2 weeks, amikacin and oral gatifloxacin 2 weeks. Followed by gatifloxacin oral. | Susceptible to Aminocycline,ciprofloxacin,gatifloxacin,clarithromycin,gentamicin,kanamycin,tobramycin,TMP-SMZ and linezolid | 7 | 7  months |
| Thoms et al,2007(Thoms et al., 2007) | Germany | 55 M | No external injuries to health | skin | Skin biopsy specimens were cultured after excision | Amikacin and imipenem for 4 weeks, TMP-SMZ 1 years | Amikacin, imipenem and TMP-SM are sensitive | Recovered | 1 year |
| Pelaez et al,2009(Pelaez et al., 2009) | Spain | 85 F | COPD | Disseminated (lung, empyema, brain) | Chest fluid culture | TMP-SMZ and imipenem 10 days occured tonic clonic seizures. Oral linezolid 1 week occured anemia and thrombocytopenia. | Susceptible to TMP-SMZ, amikacin, gentamicin, linezolid, imipenem;  Intermediary erythromycin,vancomycin;  Resistant to Penicillin | Died | One month |
| Betrán et al,2010(Betrán et al., 2010) | Spain | 57M | Diabetes, thrombocytopenia, glucocorticoids | lung | Sputum culture + gene sequencing | TMP-SMZ | Susceptible to:Aminocycline,gentamicin and TMP-SMZ | Recovered | not quite clear |
| Chen B et al,2011(Chen et al., 2011) | China | 51M | Health, trauma | Skin, soft tissue | Sputum culture and gene sequencing | TMP-SMZ | - | Recovered | 1 year |
| Ramamoorthiet al,2011(Ramamoorthi et al., 2011) | India | 36F | Healthy, no trauma | lung | Culture of lavage fluid from alveoli | TMP-SMZ | - | Recovered | 6 months |
| Shahapur et al,2014(Shahapur et al., 2014) | India | 60 years old | COPD, injury | skin | Sputum culture + gene sequencing | TMP-SMZ non-improvement. Amikacin  and linezolid 4 weeks improvement.Oral linezolid 8 weeks | Susceptible to Aminocycline,minocycline and linezolid | Recovered | 3 months |
| Jiang et al,2016(Jiang et al., 2016) | China | 37M | Immune function is normal and there is no trauma | Disseminated (neck soft tissue, lung, liver) | Cultures of neck masses | Minocycline and TMP-SMZ | Susceptible to TMP-SMZ, linezolid, minocycline and amikacin | Recovered | 4  months |
| Deepa et al. 2016(Deepa et al., 2016) | India | 14 F | rheumatic heart disease | lung | Chest water culture | Ceftriaxone, Died before diagnosis | Susceptible to gentamicin,ciprofloxacin,amikacin,TMP-SMZ, tetracycline,imipenem  Resistant to Clavulanic acid amoxicillin,cefotaxime, ceftriaxone | Died | 5 days |
| Sadamatsu et al. 2017(Sadamatsu et al., 2017) | Japan | 72 F | Asthma, glucocorticoids | lung | Sputum culture + gene sequencing | TMP-SMZ and minocycline 4weeks occured nausea,  switch to levofloxacin | Susceptible to TMP-SMZ,minocycline,imipenem/cilastatin,levofloxacin | Recovered | 6 months |
| Liu et al,2017(Liu et al., 2017) | China | 58 M | Normal immune, HBV carrier, cotton farmer, smoking history | lung | Sputum culture + mass spectrometry | TMP-SMZ amikacin imipenem,Died 7 days later due to severe dyspnea | Susceptible to TMP-SMZ,amikacin, imipenem | Died | 13 days |
| Princess et al. 2018(Princess et al., 2018) | India | 51M | Glucocorticoids for asthma | lung | Sputum culture + mass spectrometry | TMP-SMZ and imipenem | Susceptible to Amikacin,ciprofloxacin,linezolid, imipenem , ceftriaxone  Resistant to TMP-SMZ, amoxicillin/clavulanate | Died | 7 days |
| Tajima et al. 2018(Tajima et al., 2018) | Japan | 66 M | lymphadenoma | lung, brain | Blood culture + mass spectrometry | TMP-SMZ + linezolid | Susceptible to Amikacin, linezolid, minocycline,TMP-SMZ, tobramycin,doxycycline, gentamicin | Recovered | 3 months |
| Carabelli et al,2019(Carabelli et al., 2019) | Spain | 89 F | persistent asthma (ICS inhalation), bronchiectasis, obesity, normal immune function | Lung, empyema | sputum culture | TMP-SMZ, amikacin and imipenem at 20 days | Susceptible to TMP-SMZ,amikacin and imipenem | Died | 35 days |
| Paniagua-García  et al,2019(Paniagua-García et al., 2019) | Spain | 57M | diabetes mellitus, COPD | Disseminated (lung, brain, skin) | Chest water culture | TMP-SMZ + linezolid | Susceptible to Amikacin, trimethoprim,TMP-SMZ,and linezolid | Died | 1 week |
| Saksena et al,2020(Saksena et al., 2020) | India | 1) 70 F  2) 74M | 1. Immune function is normal   2) Tuberculosis was cured more than 10 years ago, smoking and drinking | 1) Lung  2) Lung | sputum culture  sputum culture | 1) TMP-SMZ1 day  2) TMP-SMZ5 days | Susceptible to Amikacin, linezolid, ciprofloxacin and gentamicin Resistant to Ampicillin, amoxicillin-clavulanate, erythromycin,TMP-SMZ and imipenem | 1) Died  2) Died | 1 day  5 days |
| Sah et al, 2020(Sah et al., 2020) | India | 61M | Nephrotic syndrome, glucocorticoids | lung, skin | Sputum culture + mass spectrometry | TMP-SMZ,meropenem,amikacin improved  TMP-SMZ was taken orally after discharge | Susceptible to TMP-SMZ,imipenem, amikacin and linezolid  Resistant to Ceftriaxone | Recovered | 7 months |
| Douedi et al,2020(Douedi et al., 2020) | America | 56M | Diabetes, PICC catheter | Soft tissue of the skin | Abscess culture | Abscess incision and drainage, PICC catheter removal, TMP-SMZ | Susceptible to TMP-SMZ,minocycline,imipenem and linezolid | Recovered | 3 months |
| Parengala et al,2021(Parengal et al., 2021) | Qatar | 29 F | SLE, glucocorticoids + immunosuppressants | Disseminated (skin + lung, brain, liver, spleen, kidney) | Sputum + alveolar lavage fluid + skin pustule culture + mass spectrometry | Meropenem,TMP-SMZ and amikacin for 8 weeks, craniotomy was performed for deterioration; moxifloxacin and TMP-SMZ were continued orally | Susceptible to tmp-smz, amikacin, ciprofloxacin,moxifloxacin and linezolid. Resistant to Amoxicillin-clavulanate,ceftriaxone and clarithromycin | Recovered | 1 year |
| Einstein et al,2021(Einstein et al., 2021) | America | 46M | Diabetes, normal immune function | lung, brain | Cerebrospinal fluid culture + mass spectrometry | Amikacin,imipenem and TMP-SMZ.Transition to oral TMP/SMZ and minocycline for1 year; lifelong secondary prophylaxis with TMP/SMZ | Susceptible to TMP/SMZ,amikacin,tobramycin and minocycline | Recovered | lifelong |
| Kullab et al,2022(Kullab et al., 2022) | Saudi Arabia, Asia | 36 F | Chemotherapy for breast cancer | Disseminated (lung, skin, brain) | Sputum culture mass spectrometry | Amikacin, linezolid and ceftriaxone. Moxifloxacin was administered after 5 weeks. | Susceptible to linezolid, amikacin;  Intermediates moxifloxacin,ceftriaxone sodium, doxycycline,minocycline;  Resistant to TMP-SMZ,ciprofloxacin, imipenem, amoxicillin/potassium clavulanate,tobramycin,clarithromycin | Recovered | 14 weeks |
| Albuni et al,2022(Albuni et al., 2022) | Qatar | 47M | Nephrotic syndrome, glucocorticoid + immunosuppressant | Skin soft tissue + lung + head | Synovial fluid culture + mass spectrometry | TMP-SMZ,moxifloxacin for 2 weeks, followed by TMP-SMZ oral | Susceptible to TMP-SMZ,linezolid,moxifloxacin  Resistant to Ceftriaxone | Recovered | 1year |
| Das et al,2022(Das and Singh, 2022) | India | 42 M | Scleroderma, glucocorticoids | brain | Sputum culture | intravenous TMP-SMZ and imipenem4weeks,followed by TMP-SMZ oral for 2 months | - | Recovered | 3 months |
| Barry et al,2022 (Barry et al., 2022) | Saudi Arabia | 37 F | Breast cancer chemotherapy, gardening soil work | Disseminated (skin, intracranial) | Brain lesions + mass spectrometry analysis | TMP-SMZ and imipenem + amikacin for22 days, changed to amikacin, linezolid, moxifloxacin and doxycycline | Resistant to TMP-SMZ and imipenem  Susceptible to Amikacin, linezolid | Died | 1 month |
| Duangprasert ,et al,2022(Duangprasert et al., 2022) | Thailand | 51M | Autoimmune hepatitis, glucocorticoid + antiviral + immunosuppressant | Lung,rupture of an infectious intracranial aneurysm | sputum culture | TMP-SMZ,amikacin and moxifloxacin. | Susceptible to TMP-SMZ,amikacin and moxifloxacin  Resistant to imipenem | Died | 5 days |
| Williamet et al,2023(William et al., 2023) | India | 62M | Tuberculosis, COVID-19, glucocorticoids | Initial skin, subsequent lung | Sputum culture | TMP-SMZ,linezolid | - | Recovered | 2 weeks |
| Fu et al,2023(Fu et al., 2023) | America | 73M | COPD | Skin, lung | Culture of lavage fluid from alveoli | TMP-SMZ, amikacin and levofloxacin.Switched to linezolid and clarithromycin, amikacin, acute kidney injury and QT interval prolongation occurred | Susceptible to Amikacin, clarithromycin,linezolid,moxifloxacin Resistant to TMP-SMZ,amoxicillin ,clavulanate potassium, ceftriaxone,doxycycline | Died | 6 weeks |
| Pérez Ramos et al, 2024(Pérez Ramos et al., 2024) | Spain | 86 F | Chronic thromboembolism, pulmonary hypertension | Lung, empyema | Chest fluid culture + gene sequencing | TMP-SMZ and linezolid intravenously,then oral tetracycline at 200 mg/24 h | Susceptible to TMP-SMZ,amikacin and linezolid.  Resistant to imipenem, amoxicillin/clavulanate and ceftriaxone | Recovered | 1 year |
| Nallamotu et al., 2024(Nallamotu and Reddy, 2024) | India | 49M | Health and immune function are normal | lung | Culture of lavage fluid from alveoli | TMP-SMZand amikacin intravenously for 2 weeks, followed by TMP-SMZ oral for 6 months | Resistant to Ampicillin/amoxicillin, cefotaxime/ceftriaxone ; Susceptible to Amikacin, amoxicillin-clavulanate,ciprofloxacin/ofloxacin,trimethoprim-sulfamethoxazole,erythromycin,gentamicin,tetracycline/doxycycline,imipenem,linezolid | Recovered | 6 months |
| Huang et al, 2024(Huang and Jia, 2024) | China | 66 F | COPD, bronchiectasis, immune function normal | Disseminated (skin, soft tissue, lung, brain) | Lobar lavage fluid ngS second generation gene sequencing | TMP-SMZ oral, imipenem intravenousfor2 weeks. TMP-SMZ oral continued for 10 months | - | Recovered | 1 year |
| Srivastava et al,2024(Srivastava et al., 2024) | India | 55 M | Old myocardial infarction, chronic kidney disease dialysis | lung | Chest fluid culture + mass spectrometry | Two weeks after TMP-SMZ the patient developed upper gastrointestinal bleeding with thrombocytopenia | - | Died | 15 days |
| Zhang et al,2024(Zhang et al., 2024) | China | 66 M | Nephrotic syndrome, glucocorticoids | Disseminated (testis, lung, skin soft tissue, intracranial, | Cerebrospinal fluid culture + mass spectrometry | Linezolid + compound sulfamethoxazole combined therapy | Susceptible to Linezolid,TMP-SMZ | Recovered | 20 days |
| Ranjan et al,2024(Ranjan et al., 2024) | India | 38M | HLH/ITP, steroids, HIV, immunodeficiency | Lung, empyema | Tongue water chromatography analysis | Ofloxacin and amikacin | Resistant to ampicillin, ceftriaxone, imipenem, co-trimoxazole,erythromycin and tetracycline. Susceptible to amikacin, linezolid and levofloxacin | Died | 40 days |
| Kanagiri1  Wait 20(Kanagiri et al., 2024)24 | India | 46 F | Four years ago, he had a history of lung nocardia + aspergillus infection, CD4 lymphocyte reduction, | lung | Alveolar lavage culture +ngs | TMP-SMZand amikacin was injected for 2 weeks. After discharge,TMP-SMZ was taken orally for 6 months, and lifelong secondary prevention | Susceptible to TMP-SMZ, amikacin and linezolid, Resistant to ceftriaxone | In 2024, it recurred and improved after medication | Lifetime prevention after 6 months |
| Erbaş et al,2024(Erbaş et al., 2024) | Turkey | 25-day-old newborn | hypogammag lobulinemia | Skin, soft tissue | Sputum culture + mass spectrometry | Linezolid for 8 days, followed by oral TMP-SMZ | - | Recovered | 12 months |
| Lai, etc., 2024(Lai et al., 2024) | China | 67M | Immune function is normal and there is no trauma | lung | foster +mngs | TMP-SMZ | - | Recovered | not quite clear |
| Cabrera-Sanchez et al,2024(Cabrera-Sanchez et al., 2024) | Peru | 51 F | Asthma,colonic adenocarcinoma with liver and brain metastases, rheumatoid arthritis, chemotherapy | lung | Cultivation + mass spectrometry analysis | Doxymethoxylate-sulfamethoxazole and imipenem/cilastatin | - | Recovered | 6 months |
| Fan et al.2024(Fan et al., 2024) | China | 78M | Health, farmers | lung | Sputum culture +ngs | Sulfamethoxazole and intravenous meropenem were used for treatment | - | Recovered | 4 months |
| Lin et al.2024(Lin et al., 2024) | China | 68M | peasant,copd | Lung, with aspergillus | foster +tngs | Sulfamethoxazole/trimethoprim, moxifloxacin, voriconazole | - | Recovered | 3 months |

1. Specific description of mngs detection

Metagenomic Testing Procedure:The necessity of removing host cells from the bronchoalveolar lavage fluid sample was determined based on the human host cell content (removal was required if >1×10⁶ cells/mL). If the sample was viscous, it was liquefied first. Cells were disrupted using a cell disruptor and then centrifuged. A volume of 600 μL of the supernatant was collected, and DNA was extracted using a nucleic acid extraction and purification kit (Genskey, Tianjin, China). The extracted nucleic acids were subjected to fragmentation, end repair, adapter ligation, PCR amplification, and magnetic bead purification using the Sequencing Reaction Prep Universal Kit (Genskey, Tianjin, China) to construct the DNA library. Library quality control was performed using an Agilent 2100 Bioanalyzer (Agilent Technologies, Santa Clara, USA) to assess library and insert fragment sizes. Library concentration was determined using quantitative real-time PCR. The constructed library was pooled and sequenced on the MGISEQ-2000 platform (MGI Tech Co., Ltd, Shenzhen, China), with a single-end read length of 50 bp (SE50).

Bioinformatics Analysis:Raw sequencing data were converted to the fq format for processing using bcl2fastq software. Low-quality, low-complexity, and short sequences were filtered out using fastp software. Human sequences were removed by aligning reads with bowtie2 against the human reference genome T2T-CHM13 (Telomere-to-Telomere Consortium Human Genome Assembly in Chromosome 13). The high-quality sequencing data obtained were aligned against a custom reference database using the BWA-MEM software tool. This database was primarily constructed from the NCBI RefSeq and GenBank genomic databases. Advanced data analysis was performed on matched sequences to generate a list of suspected pathogenic microorganisms, including information on read length and coverage.

References

Alberts, J.H., and Boyd, A.S. (2002). Nocardia otitidiscaviarum: an unusual Nocardia species causing a primary lymphocutaneous infectious process in a mildly immunosuppressed patient. *Skinmed.* 1, 62-64. doi: 10.1111/j.1540-9740.2002.01740.x.

Albuni, M.K., Kanjo, W., El Siddig Abdelrahman, H.A., Lutf, A., Al samawi, M.A., , et al. (2022). Disseminated Nocardia otitidiscaviarum presenting as septic arthritis in a nephrotic syndrome patient on immunosuppressants. *Med. case rep.study protoc.* 3, e0234. doi: 10.1097/md9.0000000000000234.

Barry, M., Alshehri, S., Alguhani, A., Barry, M., Alhijji, A., Binkhamis, K., et al. (2022). A fatal case of disseminated nocardiosis due to Nocardia otitidiscaviarum resistant to trimethoprim–sulfamethoxazole: case report and literature review. *Ann Clin Microbiol Antimicrob.* 21, 17. doi: 10.1186/s12941-022-00511-9.

Betrán, A., Villuendas, M.C., Rezusta, A., Moles, B., Rubio, M.C., Revillo, M.J., et al. (2010). Cavitary pneumonia caused by Nocardia otitidiscaviarum. *Braz J Microbiol.* 41, 329-332. doi: 10.1590/s1517-83822010000200011.

Cabrera-Sanchez, J., Tejada, H., and Ordaya, E.E. (2024). Nocardia otitidiscaviarum Pneumonia and Empyema in a Woman With Colon Adenocarcinoma. *Cureus.* 16, e72663. doi: 10.7759/cureus.72663.

Carabelli, T.S., Ronda, V.E., and Moreno, K.H. (2019). Nocardia otitidiscaviarum Infection in an Immunocompetent Patient. *Arch Bronconeumol.* 55, 59-60. doi: 10.1016/j.Arbr.2018.11.004.

Castelli, L., Zlotnik, H., Ponti, R., and Vidotto, V. (1994). First reported Nocardia otitidiscaviarum infection in an AIDS patient in Italy. *Mycopathologia.* 126, 131-136. doi: 10.1007/bf01103766.

Chen, B., Zhu, L.-y., Xuan, X., Wu, L.-j., Zhou, T.-l., Zhang, X.-q., et al. (2011). Isolation of both Pseudozyma aphidis and Nocardia otitidiscaviarum from a mycetoma on the leg. *Int J Dermatol.* 50, 714-719. doi: 10.1111/j.1365-4632.2010.04814.x.

Clark, N.M., Braun, D.K., Pasternak, A., and Chenoweth, C.E. (1995). Primary cutaneous Nocardia otitidiscaviarum infection: case report and review. *Clin Infect Dis.* 20, 1266-1270. doi: 10.1093/clinids/20.5.1266.

Das, L., and Singh, A.S. (2022). Nocardia brain abscess: a sinister aetiology. *Postgrad Med J.* 98, e30. doi: 10.1136/postgradmedj-2021-140656.

Deepa, R., Banu, S.T., Jayalakshmi, G., and Parveen, J.D. (2016). Pleuropulmonary nocardiosis due to Nocardia otitidiscaviarum in a debilitated host. *Indian J Pathol Microbiol.* 59, 240-242. doi: 10.4103/0377-4929.182011.

Dikensoy, O., Filiz, A., Bayram, N., Balci, I., Zer, Y., Celik, G., et al. (2004). First report of pulmonary Nocardia otitidiscaviarum infection in an immunocompetent patient from Turkey. *Int J Clin Pract.* 58, 210-213. doi: 10.1111/j.1368-5031.2004.0133.x.

Douedi, S., Fadhel, M., and Patel, S. (2020). Iatrogenic Nocardia otitidiscaviarum after PICC line placement. *IDCases.* 22, e00986. doi: 10.1016/j.idcr.2020.e00986.

Duangprasert, G., Kebboonkird, D., Ratanavinitkul, W., and Tantongtip, D. (2022). A rare case of ruptured anterior cerebral artery infected aneurysm with angioinvasion secondary to disseminated Nocardia otitidiscaviarum: A case report and literature review. *Surg Neurol Int.* 13, 417. doi: 10.25259/sni_734_2022.

Durán, E., López, L., Martínez, A., Comuas, F., and Rubio, M.C. (2001). Primary brain abscess with Nocardia otitidiscaviarum in an intravenous drug abuser. *J Med Microbiol.* 50, 101-103. doi: 10.1099/0022-1317-50-1-101.

Einstein, E.H., Bonda, D., Khan, S., Zlochower, A.B., and D'Amico, R.S. (2021). Multiple Brain Abscesses Due to Nocardia otitidiscaviarum: Case Report and Treatment Implications. *Cureus.* 13, e14362. doi: 10.7759/cureus.14362.

Erbaş, İ.C., Çakil Güzin, A., Özdem Alataş, Ş., Akyildiz, C., Üçüncü Egeli, T., and Belet, N. (2024). Newborn With Branchial Cleft Cyst Infection Due to Nocardia otitidiscaviarum. *Pediatr Infect Dis.* 43, e222-e224. doi: 10.1097/inf.0000000000004296.

Fabre, S., Gibert, C., Lechiche, C., Jorgensen, C., and Sany, J. (2005). Primary cutaneous Nocardia otitidiscaviarum infection in a patient with rheumatoid arthritis treated with infliximab. *J Rheumatol.* 32, 2432-2433.

Fan, N., Fang, H., Huang, F., Zhou, J., Liu, P., Li, M.-J., et al. (2024). Metagenome next-generation sequencing plays a key role in the diagnosis and selection of effective antibiotics on the treatment of Nocardia pneumonia: a case report. *Front Med (Lausanne).* 11, 1373319. doi: 10.3389/fmed.2024.1373319.

Fu, K., White, K., Ramaniuk, A., Kollu, V., and Urbine, D. (2023). Manifestations and Management of Trimethoprim/Sulfamethoxazole--Resistant Nocardia otitidiscaviarum Infection. *Emerg Infect Dis.* 29, 1266-1267. doi: 10.3201/eid2906.221854.

Hartmann, A., Halvorsen, C.E., Jenssen, T., Bjørneklett, A., Brekke, I.B., Bakke, S.J., et al. (2000). Intracerebral abscess caused by Nocardia otitidiscaviarum in a renal transplant patient--cured by evacuation plus antibiotic therapy. *Nephron.* 86, 79-83. doi: 10.1159/000045716.

Hemmersbach-miller, M., Martel, A.C., Benítez, A.B., and Sosa, A.O. (2004). Brain abscess due to Nocardia otitidiscaviarum: report of a case and review. *Scandinavian Journal of Infectious Diseases.* 36, 381-384. doi: 10.1080/00365540410020398.

Huang, L., and Jia, L. (2024). Disseminated nocardiosis caused by Nocardia otitidiscaviarum-A case report. *Diagn Microbiol Infect Dis.* 110, 116347. doi: 10.1016/j.diagmicrobio.2024.116347.

Jiang, Y., Huang, A., and Fang, Q. (2016). Disseminated nocardiosis caused by Nocardia otitidiscaviarum in an immunocompetent host: A case report and literature review. *Exp Ther Med.* 12, 3339-3346. doi: 10.3892/etm.2016.3755.

Kanagiri, T., Meena, D.S., Kumar, D., Midha, N.K., Kombade, S., and Yadav, T. (2024). Recurrent pulmonary nocardiosis due to Nocardia Otitidiscaviarum in a patient with isolated CD4 lymphocytopenia: a case report. *BMC Infect Dis.* 24, 1033. doi: 10.1186/s12879-024-09981-y.

Kullab, S., Basamad, S.W., Alnwaisir, M., Alhowari, M., and Nohuz, E. (2022). Disseminated Nocardiosis in a Breast Cancer Patient Caused by Nocardia otitidiscaviarum: A Case Report of Tertiary Centre in Saudi Arabia. *Cureus.* 14, e22686. doi: 10.7759/cureus.22686.

Lai, Y., Zhou, F., Wang, H., He, X., Zhang, Q., and Zhou, Y. (2024). Application of bronchoalveolar lavage fluid cytomorphology in diagnosing Nocardia otitidiscaviarum: a case report. *J Med Case Rep.* 18, 577. doi: 10.1186/s13256-024-04920-6.

Lin, C., Wang, S.S., An, R., Feng, T., and Huang, S.M. (2024). Pulmonary co-infection with Nocardia otitidiscaviarum and Aspergillus: a case report. *Zhonghua Jie He He Hu Xi Za Zhi.* 47, 237-240. doi: 10.3760/cma.j.cn112147-20230714-00008.

Liu, C., Feng, M., Zhu, J., Tao, Y., Kang, M., and Chen, L. (2017). Severe pneumonia due to Nocardia otitidiscaviarum identified by mass spectroscopy in a cotton farmer: A case report and literature review. *Medicine(Baltimore).* 96, e6526. doi: 10.1097/MD.0000000000006526.

Mereghetti, L., van der Mee-Marquet, N., Dubost, A.F., and Boiron, P. (1997). Nocardia otitidiscaviarum infection of a traumatic skin wound. *Eur J Clin Microbiol Infect Dis.* 16, 383-384. doi: 10.1007/bf01726368.

Nallamotu, S., and Reddy, M.S. (2024). Exposing the Masquerade of Nocardia otitidiscaviarum Pneumonia: A Case Report. *Cureus.* 16, e67849. doi: 10.7759/cureus.67849.

Paniagua-García, M., Palacios-Baena, Z.R., and Del Toro López, M.D. (2019). Disseminated nocardiosis secondary to Nocardia otitidiscaviarum infection with fatal outcome. *Med Clin.* 153, e55-e56. doi: 10.1016/j.medcli.2019.01.021.

Parengal, J., Alebbi, S.M., Hamed, M.M.M., Alqatami, H.M., and Abid, F.B. (2021). Disseminated life threatening Nocardia otitidiscaviarum infection in a young female with newly diagnosed systemic lupus erythematosus, case report and review of literature. *IDCases.* 26, e01265. doi: 10.1016/j.idcr.2021.e01265.

Pelaez, A.I., Garcia-Suarez, M.d.M., Manteca, A., Melon, O., Aranaz, C., Cimadevilla, R., et al. (2009). A fatal case of Nocardia otitidiscaviarum pulmonary infection and brain abscess: taxonomic characterization by molecular techniques. *Ann Clin Microbiol Antimicrob.* 8, 11. doi: 10.1186/1476-0711-8-11.

Pérez Ramos, I.S., Gurruchaga Yanes, M.L., Fernández Vecilla, D., Oiartzabal Elorriaga, U., Unzaga Barañano, M.J., and Díaz de Tuesta Del Arco, J.L. (2024). Cavitary pneumonia and empyema thoracis caused by multidrug resistant Nocardia otitidiscaviarum in an elderly patient. *Rev Esp Quimioter.* 37, 97-99. doi: 10.37201/req/042.2023.

Princess, I., Ebenezer, R., Ramakrishnan, N., and Nandini, S. (2018). Pulmonary Nocardiosis and Scrub Typhus in an Immunocompromised Host. *J Glob Infect Dis.* 10, 108-111. doi: 10.4103/jgid.jgid_105_17.

Ramamoorthi, K., Pruthvi, B.C., Rao, N.R., Belle, J., and Chawla, K. (2011). Pulmonary nocardiosis due to Nocardia otitidiscaviarum in an immunocompetent host- a rare case report. *Asian Pac J Trop Med.* 4, 414-416. doi: 10.1016/s1995-7645(11)60116-8.

Ranjan, R., Bir, R., Gunasekaran, J., Yadav, V.S., and Gupta, R.M. (2024). A Fatal Case of Multidrug-Resistant Pleural Nocardiosis by Nocardia otitidiscaviarum in an Immunosuppressed Patient: A Case Report and Literature Review. *Cureus.* 16, e52071. doi: 10.7759/cureus.52071.

Sadamatsu, H., Takahashi, K., Tashiro, H., Komiya, K., Nakamura, T., and Sueoka-Aragane, N. (2017). Successful treatment of pulmonary nocardiosis with fluoroquinolone in bronchial asthma and bronchiectasis. *Respirol Case Rep.* 5, e00229. doi: 10.1002/rcr2.229.

Sah, R., Khadka, S., Neupane, S., Nepal, G., Singla, S., Kumari, P., et al. (2020). Disseminated infection with *Nocardia otitidiscaviarum* in a patient under steroid therapy. *Clin Case Rep.* 8, 369-373. doi: 10.1002/ccr3.2640.

Saksena, R., Rynga, D., Rajan, S., Gaind, R., Dawar, R., Sardana, R., et al. (2020). Fatal pulmonary infection by trimethoprim-sulfamethoxazole resistant Nocardia otitidiscaviarum: report of two cases and review. *J Infect Dev Ctries.* 14, 214-222. doi: 10.3855/jidc.10169.

Sandre, R.M., and Summerbell, R.C. (1997). Disseminated Nocardia Otitidiscaviarum in a Patient with AIDS. *Can J Infect Dis.* 8, 347-350. doi: info:doi/10.1155/1997/305246.

Scheelje Carabelli, T., Esteban Ronda, V., and Hernández Moreno, K. (2019). Nocardia Otitidiscaviarum Infection in an Immunocompetent Patient. *Arch Bronconeumol.* 55, 59-60. doi: 10.1016/j.arbres.2018.03.020.

Shahapur, P.R., Peerapur, B.V., Shahapur, R.P., Honnutagi, R.M., and Biradar, M.S. (2014). Lymphocutaneous nocardiosis caused by Nocardia otitidiscaviarum: A case report and review of literature. *J Nat Sci Biol Med.* 5, 197-201. doi: 10.4103/0976-9668.127328.

Sharma, M., Gilbert, B.C., Benz, R.L., and Santoro, J. (2007). Disseminated Nocardia otitidiscaviarum infection in a woman with sickle cell anemia and end-stage renal disease. *Am J Med Sci.* 333, 372-375. doi: 10.1097/MAJ.0b013e318065ab26.

Simmons, B.P., Gelfand, M.S., and Roberts, G.D. (1992). Nocardia otitidiscaviarum (caviae) infection in a heart transplant patient presented as having a thigh abscess (Madura thigh). *J Heart Lung Transplant.* 11, 824-826. doi: 10.1097/00007890-199208000-00037.

Srivastava, S., Samaddar, A., Khan, S., Tak, V., Bohra, G.K., Sharma, D., et al. (2024). Nocardia otitidiscaviarum causing pulmonary nocardiosis: a case report and its review of the literature. *Access Microbiol.* 6, 000530.v000535. doi: 10.1099/acmi.0.000530.v5.

Suzuki, Y., Toyama, K., Utsugi, K., Yazawa, K., Mikami, Y., Fujita, M., et al. (1995). Primary Lymphocutaneous Nocardiosis Due to Nocardia otitidiscaviarum : The First Case Report from Japan. *J Dermatol.* 22, 344-347. doi: 10.1111/j.1346-8138.1995.tb03400.x.

Tajima, K., Terada, T., Okuyama, S., Akaneya, D., Hori, R., Abe, S., et al. (2018). Nocardia otitidiscaviarum meningitis in a diffuse large B-cell lymphoma patient with CD4-positive lymphocytopenia and persistent oligoclonal CD8-positive lymphocytes in the peripheral blood. *Int J Clin Exp Pathol.* 11, 455-461.

Taniguchi, H., Mukae, H., Ashitani, J., Ihi, T., Sakamoto, A., Kohno, S., et al. (1998). Pulmonary Nocardia otitidiscaviarum infection in a patient with chronic respiratory infection. *Intern Med.* 37, 872-876. doi: 10.2169/internalmedicine.37.872.

Thoms, K.-M., Zimmermann, O., Schupp, P., Thoms, S., and Emmert, S. (2007). Nocardia otitidiscaviarum: cause of long-term cutaneous abscesses on the leg of an immunocompetent man. *Arch Dermatol.* 143, 1086-1087. doi: 10.1001/archderm.143.8.1086.

Wada, A., Matsuda, S., Kubota, H., Miura, H., and Iwamoto, Y. (2002). Primary lymphocutaneous nocardiosis caused by Nocardia otitidiscaviarum. *Hand Surg.* 7, 285-287. doi: 10.1142/s021881040200114x.

William, A., Kaur, R., Rawat, D., Mendiratta, V., and Das, S. (2023). Ulceration by Nocardia Otitidiscaviarum: A case study. *Trop Doct.* 53, 293-298. doi: 10.1177/00494755231155230.

Yang, L.Y., Chan, H.L., Chen, W.J., and Kuo, T.T. (1993). Drief communications Lyrnphocutaneous nocardiosis caused by Nocardia caviae: The first case report from Asia. *J Am Acad Dermatol.* 29, 639-641. doi: 10.1016/s0190-9622(08)81870-1.

Yoshida, K., Bandoh, S., Fujita, J., Tokuda, M., Negayama, K., and Ishida, T. (2004). Pyothorax caused by Nocardia otitidiscaviarum in a patient with rheumatoid vasculitis. *Intern Med.* 43, 615-619. doi: 10.2169/internalmedicine.43.615.

Zhang, L.-Y., Wang, L., Umar, Z., Huang, Y.-H., and Gu, B. (2024). Weathering the storm: diagnosis and treatment of a life-threatening disseminated *Nocardia otitidiscaviarum* infection. *Front Cell Infect Microbiol.* 14, 1397847. doi: 10.3389/fcimb.2024.1397847.
